# Rationale and design of the cardiovascular status in patients with endogenous cortisol excess study (CV-CORT-EX): a prospective non-interventional follow-up study

**DOI:** 10.1186/s12902-020-00665-7

**Published:** 2021-01-08

**Authors:** Kristina Ehrlich, Caroline Morbach, Theresa Reiter, Peter Ulrich Heuschmann, Anke Hannemann, Martin Fassnacht, Stefan Störk, Stefanie Hahner, Timo Deutschbein

**Affiliations:** 1 Department of Internal Medicine I, Division of Endocrinology and Diabetes, University Hospital Würzburg, University of Würzburg, Oberdürrbacher Straße 6, 97080 Würzburg, Germany; 2 Department of Internal Medicine I, Division of Cardiology, University Hospital Würzburg, University of Würzburg, Würzburg, Germany; 3 Comprehensive Heart Failure Centre, University and University Hospital Würzburg, University of Würzburg, Würzburg, Germany; 4 Academic Core Lab Ultrasound-based Cardiovascular Imaging, University Hospital Würzburg, University of Würzburg, Würzburg, Germany; 5 Clinical Trial Centre, University Hospital Würzburg, University of Würzburg, Würzburg, Germany; 6 Institute of Clinical Epidemiology and Biometry, University of Würzburg, Würzburg, Germany; 7 Institute of Clinical Chemistry and Laboratory Medicine, University Medicine Greifswald, Greifswald, Germany; 8 German Centre for Cardiovascular Research DZHK, Partner Site Greifswald, University Medicine Greifswald, Greifswald, Germany; 9 Medicover Oldenburg MVZ, Oldenburg, Germany

**Keywords:** Cardiac magnetic resonance imaging, Cardiovascular status, Cushing’s syndrome, Echocardiography, Endogenous, Glucocorticoid excess, Hormones, Left ventricular hypertrophy, Metabolic profile, Quality of life

## Abstract

**Background:**

Endogenous Cushing’s syndrome (CS) results in increased cardiovascular (CV) morbidity and mortality. So far, most studies focussed on distinct disease entities rather than the integrity of the CV system. We here describe the design of the Cardiovascular Status in Endogenous Cortisol Excess Study (CV-CORT-EX), a study aiming to comprehensively investigate the health status of patients with endogenous CS (with a particular focus on CV phenotypes, biochemical aspects, quality of life, and psychosocial status).

**Method:**

A prospective non-interventional cohort study performed at a German tertiary referral centre. At the time of enrolment, patients will be categorised as: (1) newly diagnosed overt CS, (2) recurrent overt CS, (3) CS in remission, (4) presence of mild autonomous cortisol excess (MACE). The target cohorts will be *n* = 40 (groups 1 + 2), *n* = 80 (group 3), and *n* = 20 (group 4). Patients with overt CS at the time of enrolment will be followed for 12 months after remission (with re-evaluations after 6 and 12 months). At each visit, patients will undergo transthoracic echocardiography, cardiac magnetic resonance imaging, 24-h electrocardiogram, 24-h blood pressure measurement, and indirect evaluation of endothelial function. Furthermore, a standardised clinical investigation, an extensive biochemical workup, and a detailed assessment of quality of life and psychosocial status will be applied. Study results (e.g. cardiac morphology and function according to transthoracic echocardiography and cardiac magnetic resonance imaging; e.g. prevalence of CV risk factors) from patients with CS will be compared with matched controls without CS derived from two German population-based studies.

**Discussion:**

CV-CORT-EX is designed to provide a comprehensive overview of the health status of patients with endogenous CS, mainly focussing on CV aspects, and the holistic changes following remission.

**Trail registration:**

ClinicalTrials.gov (https://clinicaltrials.gov/) NCT03880513, registration date: 19 March 2019 (retrospectively registered). Protocol Date: 28 March 2014, Version 2.

## Background

Endogenous Cushing’s syndrome (CS) is a rare disease with an annual incidence of about 4 cases per 1 million inhabitants. In around 80% of cases, the pathognomonic glucocorticoid excess is due to an inappropriate secretion of adrenocorticotropic hormone (ACTH) by a pituitary adenoma, whereas ectopic and adrenal tumours (with concomitant ACTH- or cortisol-over-secretion) are responsible for 10% of cases each [1, 2].

Irrespective of the underlying aetiology, CS patients usually present with a typical clinical phenotype (including facial plethora, proximal myopathy, purple striae, and easy bruising) and suffer from various comorbidities, including relevant cardiovascular (CV) risk factors like arterial hypertension, diabetes mellitus, and dyslipidaemia [3, 4]. The latter are likely to contribute to increased CV morbidity in CS patients, as outlined by prevalences of more than 40% for myocardial hypertrophy [5, 6], up to 30% for arteriosclerosis [7–9], and up to 5% for congestive heart failure [10, 11]. Multiple studies reported an increased mortality risk even after remission of CS. Patients mainly died of CV disease, which is probably explained by their higher incidence of myocardial infarction and ischaemic stroke compared to the general population [1, 12–16].

Physical changes, associated comorbidities, and reduced life expectancy may relevantly compromise personal well-being. Consistently, it has repeatedly been shown that more than half of CS patients suffer from impaired quality of life (QoL) or neuropsychiatric disorders [17–19].

It is thus intuitive to speculate that only an overarching understanding of the underlying pathophysiological mechanisms will enable appropriate and person-centred treatment of endogenous CS. Former studies, however, were often restricted to single clinical aspects (e.g. CV, biochemical, or psychosocial features) or diagnostic tools (e.g. either echocardiography or cardiac magnetic resonance imaging (MRI) for the evaluation of cardiac morphology and function). A more comprehensive diagnostic approach would certainly be favourable.

This study was designed to systematically characterise the effects of endogenous CS primarily on CV morphology and function, but also on physical impairment, biochemistry, and psychosocial status. To serve this goal, an expansive arsenal of diagnostic tools will be applied. The findings should then be put into context with various clinical, biochemical, and psychosocial measures. Of note, in order to assess the potential reversibility of dysfunctionalities after remission of endogenous CS, patients with overt disease will undergo re-evaluation after 6 to 12 months (i.e., with overt CS at baseline and with CS in remission during follow-up). Besides, CS patients will be matched to controls without CS out of the general population. For this, two large population-based control groups derived from the general population in different parts of Germany will be used: Characteristics and Course of Heart Failure Stages A-B and Determinants of Progression (STAAB) [20] on the one hand, and Study of Health in Pomerania (SHIP) [21] on the other.

## Design and methods

### Subjects

#### Patients with endogenous Cushing’s syndrome

Patients with endogenous CS of any aetiology (i.e. pituitary adenoma, ectopic tumour, or adrenal adenoma / hyperplasia) will be considered eligible if the inclusion and exclusion criteria (given in Table 1) are fulfilled. In contrast, patients with iatrogenic CS or adrenal carcinoma will not be enrolled, as the course of the disease is expected to result in a different clinical picture and outcome.

**Table 1:** Inclusion and exclusion criteria for the CV-CORT-EX study

**Inclusion criteria:**	
• Patients with the initial, recurrent or former diagnosis of endogenous CS of any aetiology:	
- ACTH-dependent CS (i.e. due to a pituitary adenoma or an ectopic tumour)	
- ACTH-independent CS (i.e. due to an adrenal adenoma or hyperplasia)	
• Patients with MACE, defined as the combination of an adrenal mass and a pathological 1-mg-DST serum cortisol of ≥1.8 μg/dl, but noclinical evidence of overt CS	
• Age ≥ 18 years	
• Written informed consent	
**Exclusion criteria:**	
• Current glucocorticoid pharmacotherapy (except hydrocortisone substitution therapy, usually applied after surgery for CS) or systemic glucocorticoid pharmacotherapy for > 12 months within the previous 3 years	
• Structural heart disease or chronic heart failure (NYHA > II)	
• Organ disease potentially affecting cardiac function	
• ECOG status > 2	
• Former or ongoing malignant diseases (including adrenocortical cancer) treated with (potentially) cardiotoxic therapy	
• Arterial hypertension (uncontrolled despite > 3 antihypertensive drugs)	
• Pregnancy	
• Incompliance suspected or demonstrated	
• Chronic renal failure (MDRD < 60 ml/min)	

*Abbreviations*: *ACTH* adrenocorticotropic hormone, *CS* Cushing’s syndrome, *DST* dexamethasone suppression test, *ECOG* Eastern Co-operative Oncology Group, *MACE* mild autonomous cortisol excess, *MDRD* modification of diet in renal disease, *NYHA* New York Heart Association

At study start, endogenous CS may either be overt or in remission. Care will be taken that former diagnosis of CS was made according to published guidelines [22]. Cases with adrenal disease (i.e., an adenoma or hyperplasia), inadequate response (i.e., a serum cortisol of ≥1.8 μg/dl) during the 1 mg overnight dexamethasone suppression test (1-mg-DST), and no clinical features of CS will be classified as mild autonomous cortisol excess (MACE) [23].

Study-specific investigations will be performed during regular visits at the Division of Endocrinology (University Hospital Würzburg). In case of contraindications for a diagnostic procedure (e.g. metallic implants not allowing for a cardiac MRI), patients will not undergo the particular procedure but will still be able to participate in the study. The same applies for patients who are not able or not willing to undergo certain procedures (e.g. inability to perform a chair rising test due to severe myopathy).

#### Control subjects

In order to elucidate the effects of endogenous CS, results from affected patients and controls without CS will be compared (e.g. evaluating their respective cardiac morphology and function, as described by transthoracic echocardiography and cardiac MRI). For this, subjects from two German population-based studies will be matched for possible contributors (mainly age, gender, and hypertension). A matching ratio of 1:2 (patients with CS to controls without CS) will be aimed for.

Prospective Characteristics and Course of Heart Failure Stages A-B and Determinants of Progression (STAAB) cohort study is a population-based study of inhabitants aged 30 to 79 years from the general population of Würzburg (Germany) [20]. STAAB includes a representative cohort of 5000 men and women without previous history of heart failure at baseline. As the echocardiographic workup in STAAB and CV-CORT-EX will be identical (i.e., the same investigators will apply the same diagnostic tools according to standardised operating procedures), even subtle effects of glucocorticoid excess on echocardiographic parameters will hopefully be detected [20, 24].

The Study of Health in Pomerania-Trend (SHIP-Trend) is a population-based, longitudinal study conducted in North-East Germany [21]. It is based on a representative sample including adult men and women aged between 20 and 79 years from the study region. Baseline examinations were conducted in 4420 men and women between 2008 and 2012. This study will particularly be used to elucidate differences in the cardiac MRI of patients with endogenous CS and controls without the disease.

In addition, data from both population-based studies will also be used to compare the results from other diagnostics applied in the CV-CORT-EX study (e.g. from 24-h ECG, 24-h blood pressure monitoring, and questionnaire assessment).

### Methods

#### Study design and study visits

The prospective CV-CORT-EX study will be conducted at a single German tertiary referral centre (University Hospital Würzburg) and is designed as an interdisciplinary collaborative project including the Divisions of Endocrinology and Cardiology, and the Comprehensive Heart Failure Centre. CV-CORT-EX combines a cross-sectional and a longitudinal phase. Screening and recruitment of eligible patients registered with an endogenous CS at the Division of Endocrinology will be performed between October 2014 and October 2023. Each patient will be actively invited to participate and will be informed by the study physician that participation is voluntary and that termination is possible at any time. The patient will also be informed about the examinations that should be performed during the study and their potential risks. All patients will be enrolled into the cross-sectional arm and undergo comprehensive phenotyping after written informed consent. Further, patients with overt CS will also qualify for the longitudinal arm. After an intervention resulting in remission of CS (usually surgical removal of the causative tumour), patients will be followed for 12 months (with re-evaluations after 6 and 12 months, i.e. with overt CS at baseline and with CS in remission during follow-up).

Participant retention and completion of study will be promoted through various aspects: a) performance of the most relevant study investigations on a single day; b) continuous support of study participants by the same study staff member(s), including organisation of ambulatory sampling procedures and organisation of transports and hospital visits; c) early communication between study participants and study staff member(s) to schedule re-visits (applying a study calendar with all relevant dates, timelines, and deadlines for each individual). Data from patients who decide to withdraw their study consent will be excluded.

#### Objectives

CV-CORT-EX aims to comprehensively describe CV health as well as CV related comorbidities in patients with endogenous CS. In particular, our intention is to assess the association of presence and magnitude of glucocorticoid excess to the extent of left ventricular (LV) impairment. Our present data will be compared with those obtained from a control group (matched for relevant contributors like age, sex, and hypertension). Table 2 summarises primary and secondary objectives of the study.

**Table 2:** Primary and secondary study objectives

**Primary study objectives:**	
• Prevalence and magnitude of LV hypertrophy (transthoracic echocardiography)	
• Description of LV deformation characteristics (transthoracic echocardiography)	
• Quantification of LV, total heart mass, and ventricular volumes (cardiac MRI)	
• Assessment of systolic function and myocardial structural fibrosis (cardiac MRI)	
**Secondary study objectives:**	
• Cardiac morphology and function (using transthoracic echocardiography and cardiac MRI)	
• Endothelial function (using Endo-PAT2000)	
• Blood pressure (using resting and 24-h blood pressure measurement)	
• Heart rate variability profile (using resting and 24-h electrocardiogram)	
• Clinical features (waist-to-hip ratio, body mass index, bio-impedance analysis)	
• Muscle strength (chair rising test, hand grip strength)	
• Biochemistry (including hormonal, metabolic, cardiac and routine parameters)	
• Evaluation of QoL and psychosocial status (using six questionnaires):	
- Disease-specific QoL (CushingQoL, Tuebingen CD-25)	
- Generic QoL (SF-36)	
- Generic health status (EQ-5D-3L)	
- Anxiety (GAD-7)	
- Depressive and panic disorders (brief PHQ-D)	
• Comparison between patients with CS and controls without CS (i.e. from STAAB and SHIP-Trend)	
• Comparison between patients with overt CS and patients with CS in remission	

*Abbreviations*: *CS* Cushing’s syndrome, *LV* left ventricular; *MRI* magnetic resonance imaging, *QoL* quality of life, *SHIP-Trend* Study of Health in Pomerania-Trend, *STAAB* Characteristics and Course of Heart Failure Stages A-B and Determinants of Progression

#### Evaluation of medical history and definition of CV risk factors

At each study visit, a detailed standardised medical history will be recorded, particularly focusing on CV risk factors (i.e., arterial hypertension, diabetes mellitus, dyslipidaemia, smoking), other endocrine diagnoses (e.g. hypothyroidism), and the current medication. Arterial hypertension, diabetes mellitus, and dyslipidaemia will be assumed in case of specific pharmacotherapy or pathological results during the visit. Smoking status (i.e., current, former, or non-smoking) will also be recorded. In case of current or former smoking, the number of pack-years will be calculated (20 cigarettes = one pack; one pack per day for one year = one pack year).

#### Evaluation of clinical features

Patients will undergo a comprehensive physical examination by an experienced endocrinologist, including assessment of possible CS stigmata (e.g. facial plethora, purple striae, or hirsutism).

Patients’ height and weight will be measured with a Seca 200 scale with an integrated measuring rod (Seca, Hamburg, Germany). Body mass index (BMI) will be calculated, dividing body weight by the square of height in metres (kg/m^2^).

After a five-minute rest in sitting position, resting blood pressure and heart rate will be measured in triplicate with a BoSo Medicus Prestige blood pressure cuff (BoSo Medicus, Jungingen, Germany) and a 3 M Littmann Classic III stethoscope (3 M, Neuss, Germany). Subsequently, the respective means will be calculated.

A conventional 12-lead ECG will be recorded in recumbent position, using the GE KISS device (GE Healthcare, Munich, Germany).

The circumference of three body sites will be determined with a Seca 206 stretch-resistant tape (Seca, Hamburg, Germany). Patients will have to remain in standing position (with slightly raised arms) and breathe normally during the measurement procedure. Three measurements will be performed: 1) dominant upper arm (between the acromion and olecranon); 2) waist circumference (at the midpoint between the lower margin of the last palpable ribs and the top of the iliac crest); 3) hip (at the level of the widest portion of the buttocks). Subsequently, the waist-to-hip ratio (WHR) will be calculated [25].

Bio-impedance analysis (BIA) will be performed to quantify the proportion of muscle, fat, and water. For this, the patient has to remain in horizontal position for 10 min before starting the test. Two pairs of electrodes will be fixed on the dorsal side of the hand and foot of the dominant side and will then be connected to the impedance analyser BIA 2000-S (Data Input, Wedemark, Germany). Subsequently, resistance, reactance, and phase angle will be measured via computer software, thereby determining body composition (using weight, height, age and sex as additional parameters for the distinct calculations).

With respect to the longitudinal sub-study, facial photographs (frontal and profile view) will be taken from each patient to examine the development of typical CS stigmata over time.

#### Evaluation of muscle strength

The possibility of myopathy will be indirectly evaluated by measurement of hand grip strength. Patients will grasp a hydraulic Jamar handgrip dynamometer (Patterson Medical, Warrenville, IL, USA) with the arm right-angled and at maximum force. The arithmetic mean of three readings will be calculated for each hand. Limbs with known muscle dysfunction (e.g. due to a former stroke) will be excluded from the analysis.

In addition, each patient will undergo a chair rising test as described elsewhere [26]. In short, patients are asked to rise from a sitting position at a chair of 45 cm height until full knee and hip extension. The arms must be kept crossed over the chests while performing the chair rising test. Five repetitions at maximum speed should be performed starting in sitting and ending in standing position. The read-out is the time needed to execute the task, with longer durations corresponding to more severe muscle weakness and a higher risk of falls [26].

#### Evaluation of biochemical parameters

After an overnight fast of at least 8 h, participants will undergo blood sampling between 8:00 and 9:00 a.m. These samples will be used for a comprehensive biochemical evaluation (including hormonal, metabolic, cardiac, and various routine parameters). An overview of the standardised biochemical profile is provided in Table 3. All measurements will be performed at the University Hospital Würzburg.

**Table 3:** Main parameters of the standardised biochemical profile

Endocrinology	Metabolism	Cardiology	Routine laboratory
Diurnal salivary cortisol profile	Fasting glucose	Creatine kinase	Haematology ([differential] blood count)
Serum cortisol during the 1-mg-DST	HbA1c	CK-MB	Electrolytes (sodium, potassium, magnesium, calcium, chloride, phosphate)
24-h urinary free cortisol	Triglycerides	High-sensitive troponin T	Liver parameters (GOT, GPT, y-GT, alkaline phosphatase, bilirubin)
Hair cortisol	Cholesterol (total, LDL, HDL)	NTproBNP	Kidney parameters (creatinine, urea, GFR)
Basal serum cortisol	Metabolomics		hsCRP
Basal ACTH			Coagulation (Quick, INR, PTT)

*Abbreviations*: *ACTH* adrenocorticotropic hormone, *CK-MB* creatine kinase muscle-brain type, *DST* dexamethasone suppression test, *GFR* glomerular filtration rate, *GOT* aspartate aminotransferase, *GPT* alanine aminotransferase, *HDL* high-density lipoprotein, *hsCRP* high-sensitive c-reactive protein, *INR* international normalised ratio, *LDL* low-density lipoprotein, *NTproBNP* N-terminal prohormone of brain natriuretic peptide, *PTT* partial thromboplastin time, *y-GT* gamma-glutamyltransferase

Presence and remission of CS will be confirmed via evaluation of serum cortisol during the 1-mg-DST, diurnal salivary cortisol profiles (with saliva sampling at five time points throughout a day: 08.00 a.m., 12.00 a.m., 4.00 p.m., 8.00 p.m., and 11.00 p.m.), and 24-h urinary free cortisol (taking into account published criteria, e.g. [22]). Additional biosamples (i.e. urine, blood, saliva) for medical research purposes will also be collected and stored at a local biobank (Interdisciplinary Bank of Biomaterials and Data Würzburg, ibdw) [27]. For this, samples will be transferred to the ibdw within 30 min after their collection before being pseudonymised, centrifuged, aliquoted, and stored at − 80 °C (all steps will be performed according to standardised operating procedures).

#### Evaluation of quality of life and psychosocial status

To evaluate quality of life and psychosocial status, a total of six questionnaires will be handed out to the participants in printed form. Both the CushingQoL and the Tuebingen CD-25 are disease-specific questionnaires for patients with endogenous CS [28, 29]. While the CushingQoL was established for patients with endogenous CS (irrespective of the underlying aetiology), the Tuebingen CD-25 was specifically developed for patients with Cushing’s disease. Generic health status, generic QoL, anxiety, and depressive and panic disorders will be evaluated by the questionnaires EuroQoL-5D-3L (EQ-5D-3L), Short Form 36 (SF-36), Generalized Anxiety Disorder 7 (GAD-7), and brief Patient Health Questionnaire (brief PHQ-D) [30–33].

#### Transthoracic echocardiography

Detailed transthoracic echocardiography will be performed and analysed by well trained personnel, using an ultrasound machine (Vivid 9 and Vivid E95, GE, Horton, Norway) with a 3.5 MHz sector scanner according to the standardised protocol of the Comprehensive Heart Failure Centre Würzburg. Subsequently, each investigation will be reviewed blinded to the previous results. Measurements will include standard variables of myocardial morphology and function as well as more advanced readings like speckle-tracking derived strain imaging and the assessment of myocardial work.

#### Cardiac magnetic resonance imaging

Cardiac MRI scans will always be acquired on clinical 1.5 T and 3.0 T scanners (Achieva (1.5 T) and Achieva DS (3.0 T), Philips Healthcare, Best, The Netherlands). Gadobutrol (Dosage 0.15 mmol/kg, Bayer, Leverkusen, Germany) will be used as contrast agent. According to recommendations published by the Society for Cardiovascular Magnetic Resonance (SCMR), the local protocol contains CINE imaging in short and long axes, flow- measurements in the aorta ascendens and main pulmonary artery, and detection of global and focal fibrosis with late enhancement imaging and parametric mapping. Standard clinical parameters will be analysed according to the recommendations of the SCMR and all data will be stored as Digital Imaging and Communications in Medicine (DICOM) format for further post-processing [34]. The interpretation of the cardiac MRI scans will be performed by a cardiac MRI certified cardiologist and separately reviewed by a blinded experienced cardiologist with special training in cardiac MRI. Discrepant results will be decided in consensus or through involvement of a third investigator.

#### 24-h electrocardiogram and blood pressure measurement

All study participants will undergo 24-h analyses of heart rate and blood pressure. Heart rate will be recorded using the Philipps Digi Trak XT (Philipps, Hamburg, Germany). Scans will be evaluated by experienced cardiologists, and mean heart rate during day and night time as well as heart rate variability will be derived (i.e. a 24-h ECG). Blood pressure will be recorded using a Custo screen 300 (custo med, Ottobrunn, Germany) blood pressure cuff applied to the left upper arm. Blood pressure measurements will be taken every 15 min (between 6.00 a.m. and 10.00 p.m.) or every 30 min (between 10.00 p.m. and 6.00 a.m.). Mean blood pressure during day and night time and dipping status will be evaluated.

#### Evaluation of endothelial function

The Endo-PAT2000 device (Itamar medical, Israel) offers non-invasive evaluation of endothelial function and will be used according to the manufacturer’s instructions. A blood pressure cuff will be attached to the non-dominant arm and probes to each index finger. After a preparatory phase of 6 minutes, the arterial blood flow into the arm will be interrupted via the blood pressure cuff. After 5 minutes of hypoxemia the blood pressure cuff will be removed to re-establish blood circulation. Vasodilatation will be assessed via measurement of increasing pulse wave deflection (peripheral arterial tone, PAT) via an integrated plethysmograph. Endothelial function will be assessed via the reactive hyperaemia index defined as ratio of the post-to-pre occlusion PAT amplitude of the occluded side, divided by the post-to-pre occlusion ratio of the control side. Arterial stiffness will be evaluated via the augmentation index quantified via pulse waveform analysis of the PAT signal and normalised to a heart rate of 75 bpm.

#### Data collection and management

Source data are the electronically generated original results of technical examinations, including biochemical analyses. The responsible study team (including physicians and nurses) will enter all collected data into case report forms (CRFs). Missing investigations or data in the CRFs will be flagged, along with a statement on the specific reason. Corrections will be made in accordance with Good Clinical Practice (GCP) guidelines, i.e. the version to be corrected will be crossed out so that it is still legible. The corrections (or other entries) will be marked with the date, hand sign and, if necessary, the reason for the changes conducted by the investigator. Incorrect entries will not be covered with correction fluid, erased, or otherwise rendered illegible. Finally, all data will be transferred into an excel file by the responsible study team. The investigator will maintain a patient identification list which will enable the unique assignment of patient numbers to patient name, date of birth, and sex.

#### Sample size

Due to the rarity of the disease and the paucity of literature, reliable data on cardiac morphology and function in patients with endogenous CS are lacking. Accordingly, this is an explorative study only (i.e. without formal calculation of case numbers), and the collected data will be used for a post-hoc power analysis. The estimated number of participants is *n* = 140 patients. The target cohorts will be *n* = 40 (for patients with newly diagnosed or recurrent overt CS), *n* = 80 (for patients with CS in remission), and *n* = 20 (for patients with MACE).

#### Statistical considerations

Results will be interpreted on the basis of a complete-case analysis. Missing data or investigations that cannot be interpreted will be excluded. Distribution of data will be evaluated by the Shapiro-Wilk test and quantile-quantile plots, as appropriate. Data will be reported by mean (SD), median (quartiles), and count (percent), as appropriate. Χ^2^-test, Mann-Whitney U-test or Kruskal-Wallis test will serve to compare groups. Changes from baseline to follow-up will be assessed using Wilcoxon’s signed-rank test. Univariable associations will be analysed with the Spearman’s rho coefficient. ANCOVA models and multivariable linear regression models will be applied to examine the impact of cofactors or potential confounders on crude or change variables.

## Discussion

Endogenous CS is associated with various CV risk factors leading to increased CV morbidity and mortality [2]. So far, studies reporting on CV outcome in patients with endogenous CS primarily focused on particular pathogenic aspects, applied single diagnostic tools, and had a cross-sectional rather than a longitudinal approach. The CV-CORT-EX study aims for a comprehensive CV, biochemical, and psychosocial workup of a representative number of cases with endogenous CS.

Arterial hypertension is quantitatively the most important modifiable risk factor for premature CV disease [35, 36]. It is present in up to 85% of patients with CS, and these numbers are independent of the underlying CS subtype [36]. As endogenous CS is also associated with other relevant risk factors for the development of atherosclerosis (e.g. diabetes mellitus, dyslipidaemia, chronic inflammation, endothelial dysfunction) [4, 35, 37–39], it is no surprise that an increased intima media thickness of the common carotid (as an indicator of atherosclerotic damage) has already been described in such cohorts [7, 9].

Accordingly, non-selected screening for atherosclerosis appears reasonable in case of endogenous CS. Ultrasound (e.g. of the common carotid) is observer-dependent and rather time-consuming. The fact that endothelial dysfunction is known as an early indicator of cardiovascular disease is the rationale for the use of Endo-PAT2000 device in the CV-CORT-EX study. Of note, progression of atherosclerotic plaques over time (with the consequence of slow luminal narrowing) may affect not only peripheral, but also coronary and cerebral arteries [37, 40]. Consequently, if multidetector computerised tomographic coronary angiography was applied to a cohort of CS patients, significantly more (non-) calcified coronary plaques than in a control group were observed [41].

Previous echocardiographic studies already described systolic and diastolic dysfunction as well as structural changes of the heart (particularly LV hypertrophy) in patients with endogenous CS [42–45]. Myocardial fibrosis, and reduced systolic and diastolic performance were more frequent in CS than in hypertensive controls (matched for LV hypertrophy) and healthy subjects [46]. Of note, at least two studies clearly illustrate the benefits of cardiac MRI to detect potential cardiac pathologies that are not discovered by common echocardiography [47, 48]. As a result, our aim for a combined diagnostic integration of myocardial and valvular functional status (as assessed by echocardiography) and information on cardiac morphology and myocardial fibrosis (as assessed by cardiac MRI) appears reasonable.

After remission of CS, several studies reported a reduced frequency of hypertension [9, 14, 49] on the one hand, and LV hypertrophy as well as diastolic dysfunction on the other [44, 45, 47, 48]. The same was true for CV risk factors [8, 9] and regression of myocardial fibrosis [46]. Nevertheless, the prevalence of these alterations were usually still higher compared to healthy controls [43, 45]. Besides, an increased prevalence of coronary arterial disease despite long-term remission was particularly noted for women and younger patients [50].

Although much less frequent than other CV impairments, pathological ECG findings (e.g. arrhythmias) have also been attributed to endogenous CS. In detail, irregular sinus rhythm, inverted T waves, signs of biventricular (particularly left) hypertrophy, prolonged QT, and a higher risk of impaired heart rate variability were reported, suggesting a cardiotoxic effect of excessively elevated glucocorticoids per se [36, 51–53].

Proximal myopathy is a relevant clinical finding that is present in about 40–80% of patients with overt CS [13, 54, 55]. Interestingly, while some reported a higher prevalence of myopathy in males [56], others did not find any sex-related differences [54, 55].

A higher risk of psychiatric illness (e.g. depression, anxiety, cognitive dysfunction) and a pronounced impairment of QoL have also been observed during overt CS [17–19, 57, 58]. Obviously, this is not only due to incriminatory phenotypical changes and various comorbidities, but also to effects of glucocorticoids itself [59–62]. Interestingly, female sex [28, 63, 64] and hypopituitarism [63] were described as predictors of impaired QoL. Despite remission of CS and adequate therapy of psychiatric comorbidity, persisting or sometimes even deteriorating neuropsychiatric disorders and QoL have been described [58–60, 65]. Of note, some studies even described suicidal tendencies or suicides in (currently or formerly) affected CS patients [17, 57]. Taken together, a broad assessment of quality of life and psychiatric affection appears worthwhile.

In summary, adequate treatment (both of the glucocorticoid excess and of its associated comorbidities) and continuous interdisciplinary support are required for a satisfactory long-term outcome of patients with endogenous CS. In the long-term, the CV-CORT-EX trial will hopefully pave the way for improved medical care in the future.

## Data Availability

Not applicable.

## Abbreviations

ACTH: Adrenocorticotropic hormone
BIA: Bio-impedance analysis
Brief PHQ-D: Brief Patient Health Questionnaire
CK-MB: Creatine kinase muscle-brain type
CS: Cushing’s syndrome
CV: Cardiovascular
CV-CORT-EX: Cardiovascular Status in Endogenous Cortisol Excess Study
DICOM: Digital Imaging and Communications in Medicine
DST: Dexamethasone suppression test
ECOG: Eastern Co-operative Oncology Group
ECG: Electrocardiography
EQ-5D-3L: EuroQoL-5D-3L
GCP: Good Clinical Practice
GAD-7: Generalized Anxiety Disorder 7
GFR: Glomerular filtration rate
GOT: Aspartate aminotransferase
GPT: Alanine aminotransferase
HDL: High-density lipoprotein
hsCRP: High-sensitive c-reactive protein
ibdw: Interdisciplinary Bank of Biomaterials and Data Würzburg
INR: International normalised ratio
LDL: Low-density lipoprotein
LV: Left ventricular
MACE: Mild autonomous cortisol excess
MDRD: Modification of diet in renal disease
MRI: Magnetic resonance imaging
NTproBNP: N-terminal prohormone of brain natriuretic peptide
NYHA: New York Heart Association
PAT: Peripheral arterial tone
PTT: Partial thromboplastin time
QoL: Quality of life
STAAB: Characteristics and Course of Heart Failure Stages A-B and Determinants of Progression
SCMR: Society for Cardiovascular Magnetic Resonance
SHIP: Study of Health in Pomerania
SF-36: Short Form 36
WHR: Waist-to-hip ratio
y-GT: Gamma-glutamyltransferase
